# Sex Differences in Clinical Characteristics and Prognosis in Primary Thrombotic Antiphospholipid Syndrome

**DOI:** 10.3389/fcvm.2022.895098

**Published:** 2022-07-04

**Authors:** Yongfa Huang, Huazhen Liu, Wanting Qi, Le Du, Mengtao Li, Xiaofeng Zeng, Xiaoxiao Guo, Jiuliang Zhao, Shuyang Zhang

**Affiliations:** ^1^Department of Cardiology, Peking Union Medical College Hospital, Peking Union Medical College & Chinese Academy of Medical Sciences, Beijing, China; ^2^Department of Rheumatology, Peking Union Medical College Hospital, Peking Union Medical College & Chinese Academy of Medical Sciences, Beijing, China

**Keywords:** primary thrombotic antiphospholipid syndrome, sex difference, lupus anticoagulant, thromboembolic recurrence, prognosis

## Abstract

**Objectives:**

This study aimed to investigate whether there are sex differences in clinical characteristics and prognosis in patients with primary thrombotic antiphospholipid syndrome (ptAPS).

**Methods:**

From January 2013 to July 2021, 154 consecutive patients diagnosed with ptAPS were prospectively recruited. Multivariable Cox regression was used to evaluate the association between gender and the composite endpoint including thromboembolic recurrence or all-cause death during follow-up.

**Results:**

Totally, 80 (52%) male and 74 (48%) female patients with ptAPS were included, and men had a higher percentage of smokers/ex-smokers [50 (62%) vs. 6 (8%), *p* < 0.001] and hyperhomocysteinemia [26 (32%) vs. 9 (12%), *p* = 0.003]. The baseline thromboembolic events were similar in two genders, except for limb ischemia [15 (19%) in men vs. 1 (1%) in women, *p* < 0.001]. During a median follow-up of 42 months, the composite endpoint occurred in 30 (38%) male and 15 (20%) female patients (*p* = 0.019). Male gender [HR 2.499, 95% CI (1.316, 4.743), *p* = 0.005] and warfarin administration [HR 0.482, 95% CI (0.257, 0.905), *p* = 0.023] remained independent risk factors for the composite endpoint. Male gender [HR 3.699, 95% CI (1.699, 8.246), *p* = 0.001] and isolated lupus anticoagulant positivity [HR 2.236, 95% CI (1.039, 4.811), *p* = 0.040] were independent risk factors for thromboembolic recurrence.

**Conclusion:**

There are sex disparities in the clinical characteristics in patients with ptAPS and the male gender is an independent risk factor for the poor prognosis. Male patients with isolated lupus anticoagulant (LA) positivity have the highest risk of thromboembolic recurrence.

## Introduction

Antiphospholipid syndrome (APS) is a systemic autoimmune disease featuring thromboembolic events, placental dysfunction, or recurrent fetal loss with persistent laboratory evidence of antiphospholipid antibodies (aPLs) (1, 2). APLs, principally including lupus anticoagulant (LA), anticardiolipin antibodies (aCL), and anti-β2-glycoprotein I antibodies (anti-β2-GP1), served as both diagnostic markers and pathogenic contributors in APS and had certain prognostic values for thrombotic events and relapse in different populations (3, 4). APS occurs either in the setting of an underlying disease, such as systemic lupus erythematosus (SLE) or as a primary condition that has unique mechanisms and manifestations (5, 6). Primary APS can be further classified into two categories according to the type of clinical manifestations, including isolated obstetric primary APS and primary thrombotic APS (ptAPS).

Up till now, gender effect has been recognized in epidemiology, clinical features, and pathogenesis in many autoimmune diseases, but previous studies reported few differences between the two genders in primary APS, especially in the ptAPS subpopulation (7–10). Despite the anticoagulation therapy, patients with ptAPS develop thromboembolic recurrence that could be disabling or even lethal, so it is of critical importance to identify ptAPS patients with a high risk of thromboembolic recurrence or mortality (11–13). In order to address these issues, we conducted a prospective longitudinal study of patients with ptAPS and aimed to explore gender-related clinical and prognostic differences.

## Materials and Methods

### Patient Recruitment

We conducted a prospective cohort study at Peking Union Medical College Hospital (PUMCH), Beijing, China. Patients with APS admitted to inpatient departments in PUMCH were screened by two physicians independently, who reconfirmed the diagnosis of ptAPS according to the 2006 revised Sydney criteria (1). The included patients should have had at least two positive aPL test results with an interval of at least 12 weeks. Primary APS patients with both obstetric and thrombotic events were included. Patients were excluded if complications such as systemic rheumatic disease, malignancy, or other circumstances lead to thrombophilia at the time of diagnosis or during follow-up. Herein, systemic rheumatic diseases included SLE, rheumatoid arthritis, spondyloarthropathies, systemic sclerosis, idiopathic inflammatory myopathies, undifferentiated connective tissue disease, mixed connective tissue disease, overlap syndromes, and systemic vasculitides. The study protocol was censored and approved by the Institutional Review Board of PUMCH (No. JS-1374), was conducted in accordance with the principles of the Declaration of Helsinki, and followed the International Conference on Harmonization Guideline for Good Clinical Practice. Written informed consent was obtained from all patients.

### Data Collection

Data were collected during hospital admission and outpatient follow-up, including demographics, course of the disease, laboratory tests, medications, and outcomes. Disease duration at diagnosis was calculated from the first definite ptAPS-related thromboembolic event till the first aPL positivie result, and time to thromboembolic recurrence was calculated from the onset of the ptAPS-related thromboembolic event till the first recurrent event after treatment initiation. Obesity was defined as body mass index > 30 kg/m^2^, and overweight was defined as 25 kg/m^2^ < body mass index < 30 kg/m^2^ (14). The ultrasound or computed tomography angiography images were carefully examined to distinguish between thromboembolic and vascular wall lesions. Only symptomatic stroke or transient ischemic attack (TIA) was calculated, and cardiac valve involvement included aseptic valvular vegetations or significant thickening, which could not be explained by other cardiac diseases. APS nephropathy was defined as compromised glomerular filtration, persistent hematuria or proteinuria without thrombosis in major renal blood vessels, or other potential causes (15). The composite endpoint was met if one had recurrent venous or arterial thromboembolism or died from any cause during follow-up.

The presence of aCL and anti-β2-GP1 was detected with standardized enzyme-linked immunosorbent assay using kits from Aesku Diagnostics, Germany and INOVA, United States. The cutoff values for aPLs positivity were determined with mean + 2 times the standard deviation of healthy controls, as suggested in the 2006 revised Sydney criteria (1). LA was detected with dilute Russell viper venom time/activated partial thromboplastin time, and a ratio over 1.2 was defined as positive. Anti-nuclear antibody (ANA) was detected with an indirect immunofluorescence assay (Euroimmun, Germany), and a titer greater than or equal to 1:80 was considered positive. Serum concentrations of complement and immunoglobulin were determined with immunoturbidimetry (Beckman Colter, United States).

### Statistical Analysis

Scale variables were described as medians (1st quartile, 3rd quartile), and nominal variables were described as n (%). Comparisons of scale variables were performed by the Mann–Whitney U-test. Comparisons of nominal variables between groups were performed by Pearson’s χ^2^ test or Fisher’s exact test when any cell of the contingency table contained fewer than five subjects. The association of demographics, clinical characteristics, and treatment strategies with the endpoints was identified by univariable Cox regression. Parameters with the potential prognostic value from univariable analysis (*p* < 0.1) and medical knowledge were included in the forward stepwise multivariable Cox regression, with *p* < 0.05 by the likelihood ratio test as the entry criterion and *p* > 0.10 as the removal criterion. Event-free survival and recurrence-free rate of different subgroups were assessed by the Kaplan–Meier analysis with the log-rank test. Two-sided *p*-values < 0.05 were considered statistically significant, and *p* < 0.05, *p* < 0.01, and *p* < 0.001 were marked with single, double, or triple asterisks. All statistical analyses were conducted with Prism (version 9.3.1, Graphpad Software) and SPSS Statistics (version 26.0, IBM, New York, NY, United States).

## Results

### Demographic Data and Cardiovascular Comorbidities

**FIGURE 1:**
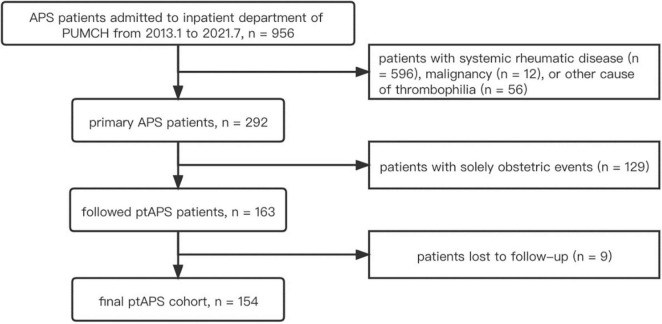
A flowchart of patient screening. APS, antiphospholipid syndrome; PUMCH, Peking Union Medical College Hospital; ptAPS, primary thrombotic antiphospholipid syndrome.

From January 2013 to July 2021, a total of 956 patients with APS were prospectively screened, and a total of 154 patients with ptAPS [80 (52%) men and 74 (48%) women] were enrolled (Figure 1). The median disease duration at diagnosis was 2 (0, 7) months for male and 1 (1, 12) month for female patients with ptAPS, and the longest disease duration at diagnosis was 51 months (Table 1). Male patients with ptAPS were more frequently complicated with traditional cardiovascular risk factors than female patients with ptAPS, including smoking [50 (62%) vs. 6 (8%), *p* < 0.001] and hyperhomocysteinemia [26 (32%) vs. 9 (12%), *p* = 0.003]. However, the incidence of atherosclerosis was comparative between the two genders in patients with ptAPS [17 (21%) vs. 10 (14%), *p* = 0.207].

**TABLE 1:** Demographic characteristics, treatment, and follow-up of patients with primary thrombotic antiphospholipid syndrome (ptAPS).

	Total	Male	Female	*P* value
N	154	80	74	–
Age of onset, years	36 (27, 51)	34 (26, 51)	36 (28, 50)	0.820
Disease duration, months	1 (1, 10)	2 (0, 7)	1 (1, 12)	0.316
Cardiovascular risk factors				
Body mass index, kg/m^2^	23.70 (21.41, 26.80)	24.78 (22.31, 27.45)	23.24 (20.72, 26.66)	0.023*
Overweight, n (%)	43 (28%)	26 (32%)	17 (23%)	0.188
Obesity, n (%)	15 (9.7%)	9 (11%)	6 (8%)	0.511
Smoking, n (%)	56 (36%)	50 (62%)	6 (8%)	<0.001***
Hypertension, n (%)	34 (22%)	22 (28%)	12 (16%)	0.092
Diabetes mellitus, n (%)	10 (6%)	6 (8%)	4 (5%)	0.748
Dyslipidaemia, n (%)	18 (12%)	12 (15%)	6 (8%)	0.184
Hyperhomocysteinemia, n (%)	35 (23%)	26 (32%)	9 (12%)	0.003**
Atherosclerosis, n (%)	27 (18%)	17 (21%)	10 (14%)	0.207
Treatment strategies				
Aspirin, n (%)	51 (33%)	28 (35%)	23 (31%)	0.606
Warfarin, n (%)	106 (69%)	55 (69%)	51 (69%)	0.982
Direct oral anticoagulants, n (%)	29 (19%)	14 (18%)	15 (20%)	0.660
Antiplatelet plus anticoagulant, n (%)	38 (25%)	19 (24%)	19 (26%)	0.782
Corticosteroid, n (%)	31 (20%)	18 (22%)	13 (18%)	0.446
Hydroxychloroquine, n (%)	84 (54%)	42 (52%)	42 (57%)	0.596
Immunosuppressants, n (%)	20 (13%)	10 (12%)	10 (14%)	0.852
Median follow-up time, months	42 (23, 67)	40 (18, 61)	48 (24, 80)	0.165
Composite endpoint, n (%)	45 (31%)	30 (38%)	15 (20%)	0.019*
Recurrent thromboembolism, n (%)	35 (23%)	27 (34%)	8 (11%)	0.001***
Venous events, n (%)	21 (14%)	16 (20%)	5 (7%)	0.019*
Deep vein thrombosis, n (%)	6 (4%)	4 (2%)	2 (3%)	0.683
Pulmonary embolism, n (%)	15 (10%)	12 (15%)	3 (4%)	0.029*
Portal vein thrombosis, n (%)	2 (1%)	1 (1%)	1 (1%)	1.000
Intracranial venous thrombosis, n (%)	2 (1%)	2 (2%)	0	0.497
Arterial events, n (%)	16 (10%)	12 (15%)	4 (5%)	0.065
Stroke/TIA, n (%)	8 (5%)	5 (6%)	3 (4%)	0.721
Myocardial infarction, n (%)	5 (3%)	3 (4%)	2 (3%)	1.000
Limb ischemia, n (%)	2 (1%)	2 (1%)	0	0.497
Adrenal infarction, n (%)	2 (1%)	2 (1%)	2 (1%)	0.497
Death, n (%)	18 (12%)	8 (10%)	10 (14%)	0.498

TIA: transient ischemic attack. *0.01 ≤ p < 0.05, **0.001 ≤ p < 0.01, and ***p < 0.001.

### Baseline Clinical Characteristics

Baseline thromboembolic events were classified into arterial and venous categories according to the location (Table 2). Totally, more venous events (67%) were reported than arterial events (48%) in patients with ptAPS. Of note, limb ischemia was more prevalent in male patients with ptAPS than their female counterparts [15 (19%) vs. 1 (1%) *p* < 0.001], and cardiac valve involvement was less common in male than female patients with ptAPS [1 (1%) vs. 7 (9%), *p* = 0.029]. Isolated LA positivity was more commonly found in male than in female patients with ptAPS, but the difference was not significant [16 (20%) vs. 7 (9%), *p* = 0.067]. Double positivity was less common in male than in female patients with ptAPS [13 (16%) vs. 27 (36%), *p* = 0.004]. ANA positivity was present in 56 (36%) patients, which was comparable between male and female patients. Some differences were observed in the serum level of IgM, C3, C4, and C-reactive protein between male and female patients, but these items were largely within reference ranges from a normal population.

**TABLE 2:** Baseline primary thrombotic antiphospholipid syndrome (ptAPS)-related events and laboratory findings.

	Total	Male	Female	*P* value
Venous thromboembolic events, n (%)	103 (67%)	53 (66%)	50 (68%)	0.862
Deep vein thrombosis, n (%)	67 (44%)	39 (49%)	28 (38%)	0.172
IVC thrombosis, n (%)	7 (5%)	3 (4%)	4 (5%)	0.711
Pulmonary embolisms, n (%)	55 (36%)	29 (36%)	26 (35%)	1.000
CTEPH, n (%)	19 (12%)	8 (10%)	11 (15%)	0.359
Portal vein thrombosis, n (%)	8 (5%)	2 (2%)	6 (8%)	0.155
Budd-Chiari syndrome, n (%)	4 (3%)	0	4 (5%)	0.051
Intracranial venous thrombosis, n (%)	9 (6%)	4 (5%)	5 (7%)	0.739
Arterial thromboembolic events, n (%)	74 (48%)	40 (50%)	34 (46%)	0.615
Stroke/TIA, n (%)	42 (27%)	19 (24%)	23 (31%)	0.307
Myocardial infarction, n (%)	17 (11%)	10 (12%)	7 (9%)	0.547
Abdominal aorta thrombosis, n (%)	7 (5%)	5 (6%)	2 (3%)	0.445
Splenic infarction, n (%)	6 (4%)	4 (5%)	2 (3%)	0.683
Renal artery thrombosis, n (%)	7 (5%)	3 (4%)	4 (5%)	0.711
Adrenal infarction, n (%)	1 (0.6%)	1 (1%)	0	1.000
Mesenteric artery thrombosis, n (%)	2 (1%)	1 (1%)	1 (1%)	1.000
Limb ischemia, n (%)	16 (10%)	15 (19%)	1 (1%)	<0.001***
Livedo reticularis, n (%)	5 (3%)	1 (1%)	4 (5%)	0.196
Cardiac valve involvement, n (%)	8 (5%)	1 (1%)	7 (9%)	0.029*
Hemolytic anemia, n (%)	16 (10%)	8 (10%)	8 (11%)	0.869
Thrombocytopenia, n (%)	31 (20%)	17 (21%)	14 (19%)	0.719
APS nephropathy, n (%)	11 (7%)	5 (6%)	6 (8%)	0.759
Retinal involvement, n (%)	10 (6%)	5 (6%)	5 (7%)	1.000
CAPS, n (%)	5 (3%)	3 (4%)	2 (3%)	1.000
aPLs categories				
aCL, n (%)	103 (67%)	52 (65%)	51 (69%)	0.606
anti-β2GP1, n (%)	122 (79%)	61 (76%)	61 (82%)	0.345
LA, n (%)	119 (77%)	64 (80%)	55 (74%)	0.401
Single positive, n (%)	39 (25%)	25 (31%)	14 (19%)	0.079
Isolated aCL, n (%)	2 (1%)	0	2 (3%)	0.229
Isolated anti-β2GP1, n (%)	14 (9%)	9 (11%)	5 (7%)	0.407
Isolated LA, n (%)	23 (15%)	16 (20%)	7 (9%)	0.067
Double positive, n (%)	40 (26%)	13 (16%)	27 (36%)	0.004**
aCL + anti-β2GP1, n (%)	19 (12%)	7 (9%)	12 (16%)	0.159
anti-β2GP1 + LA, n (%)	14 (9%)	3 (4%)	11 (15%)	0.023*
aCL + LA, n (%)	7 (5%)	3 (4%)	4 (5%)	0.711
Triple positive, n (%)	75 (49%)	42 (52%)	33 (45%)	0.327
ANA positive, n (%)	56 (36%)	24 (30%)	32 (43%)	0.088
ESR, mm/h (NR 0–15)	14 (7, 53)	13 (5, 53)	16 (7, 53)	0.473
C-reactive protein, mg/L (NR 0–8.00)	2.31 (0.85, 12.50)	5.11 (1.00, 18.82)	1.33 (0.51, 9.68)	0.016*
IgG, g/L (NR 7.00–17.00)	10.54 (8.93, 13.17)	10.51 (8.48, 13.17)	10.73 (9.29, 13.18)	0.395
IgA, g/L (NR 0.70–4.00)	1.86 (1.32, 2.62)	1.90 (1.25, 2.69)	1.86 (1.37, 2.39)	0.835
IgM, g/L (NR 0.40–2.30)	1.18 (0.91, 1.60)	1.08 (0.85, 1.41)	1.31 (1.01, 1.87)	0.008**
C3, g/L (NR 0.730–1.460)	1.089 (0.902, 1.223)	1.114 (1.000, 1.256)	1.014 (0.736, 1.171)	0.010**
C4, g/L (NR 0.100–0.400)	0.179 (0.130, 0.239)	0.201 (0.139, 0.254)	0.169 (0.112, 0.217)	0.042*

IVC: inferior vena cava; CTEPH: chronic thromboembolic pulmonary hypertension; TIA: transient ischemic attack; CAPS: catastrophic antiphospholipid syndrome; aPLs: antiphospholipid autoantibodies; aCL: anticardiolipin antibody; anti-β2GP1: anti-beta 2 glycoprotein I antibody; LA: lupus anticoagulant; ANA: anti-nuclear antibody; ESR: erythrocyte sedimentation rate; NR, normal range. *0.01 ≤ p < 0.05, **0.001 ≤ p < 0.01, and ***p < 0.001.

### Treatment Strategies and Outcomes

No difference in anticoagulation therapy or immunomodulatory treatment was observed between male and female patients with ptAPS (Table 1). During a median follow-up of 42 (23, 67) months, 45 (29%) patients with ptAPS reached the composite endpoint, including 30 (38%) male and 15 (20%) female patients (*p* = 0.019) (Table 1). Totally, 8 (10%) male patients and 10 (14%) female patients died during follow-up. Among them, 7 patients died of thromboembolic recurrence, and 5 patients died of infectious diseases. Thromboembolic recurrence was more common in male than female patients with ptAPS [27 (34%) vs. 8 (11%), *p* = 0.001]. Among all these events, the recurrence rate of pulmonary embolism (PE) was significantly higher in male than in female patients with ptAPS [12 (15%) vs. 3 (4%), *p* = 0.029]. The comparison regarding demographics, treatment, and aPLs between ones that met the composite endpoint and others were performed (Supplementary Table 1). Except for sex differences, no significant difference between the two groups was observed.

**FIGURE 2:**
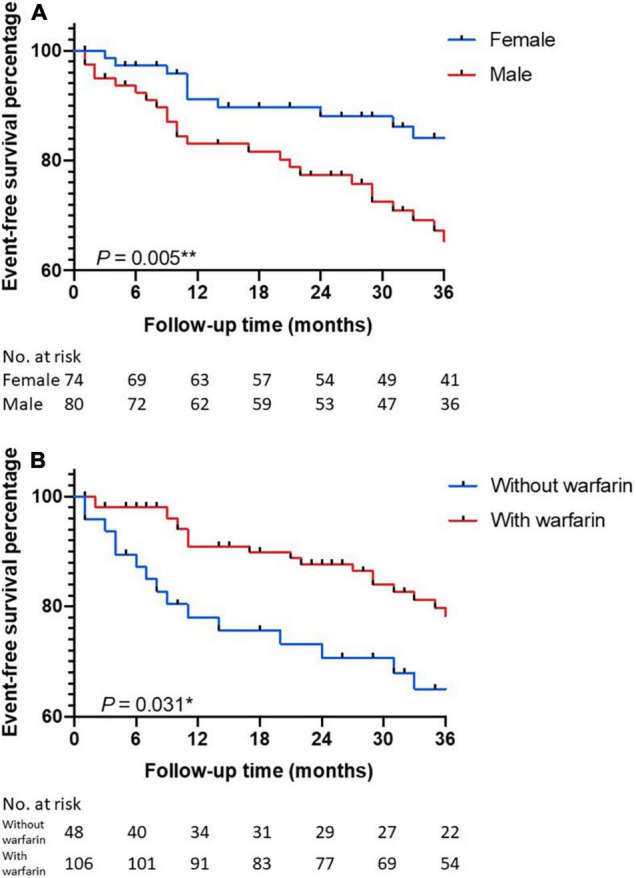
The Kaplan–Meier curves for event-free survival. Groups were defined by **(A)** gender and **(B)** warfarin administration.

**FIGURE 3:**
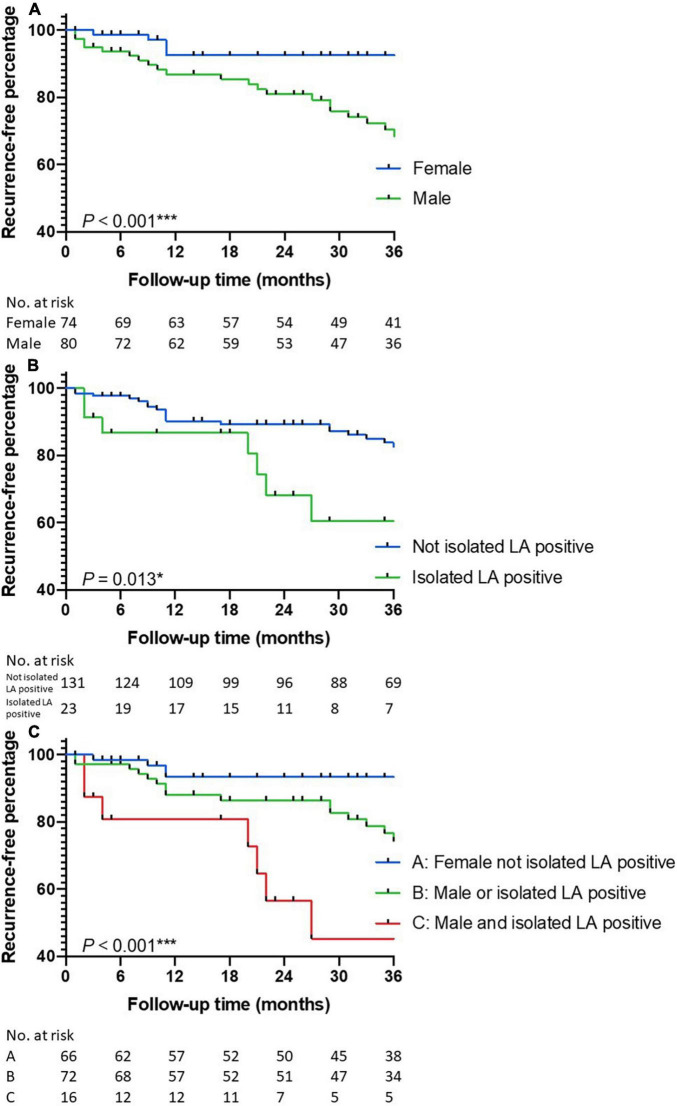
The Kaplan–Meier curves for thromboembolic recurrence. Groups were defined by **(A)** gender, **(B)** isolated LA positivity, and **(C)** risk stratification by gender and isolated LA positivity. LA, lupus anticoagulant.

Multivariable Cox regression identified male gender [HR 2.499, 95% CI (1.316, 4.743), *p* = 0.005] and warfarin administration [HR 0.482, 95% CI (0.257, 0.905), *p* = 0.023] as independent risk factors for the composite endpoint (Table 3). Male patients with ptAPS had a 2.342-fold [95% CI (1.288, 4.258), *p* = 0.005] increased risk for the composite endpoint than the female ones, and patients not treated with warfarin had a 2.160-fold [95% CI (1.075, 4.343), *p* = 0.031] increased risk for the composite endpoint against others (Figures 2A,B). The positive correlation between the male gender and the composite endpoint was mainly related to a higher risk of thromboembolic recurrence in the male group, since the male gender failed to present as a risk factor for all-cause death [HR 1.025, 95% CI (0.390, 2.695), *p* = 0.960]. Considering thromboembolic recurrence alone, male gender [HR 3.699, 95% CI (1.659, 8.246), *p* = 0.001] and isolated LA positivity [HR 2.236, 95% CI (1.039, 4.811), *p* = 0.040] were identified as independent risk factors (Table 4). During the first 3 years, the average annual rates of thromboembolic recurrence were 10.5% [95% CI (7.2%, 14.9%)] in male and 2.5% [95% CI (1.1%, 5.7%)] in female patients according to the Kaplan–Meier analysis (*p* < 0.001, Figure 3A). The average annual recurrence rates for ptAPS patients with and without isolated LA positivity were 13.1% [95% CI (6.8%, 22.2%)] and 7.9% [95% CI (5.6%, 10.9%)], respectively (*p* = 0.013, Figure 3B). The patients were further divided into 3 groups: group A (low recurrence risk) featuring female gender without isolated LA positivity, group B (medium recurrence risk) featuring male gender or isolated LA positivity, and group C (high recurrence risk) featuring male gender and isolated LA positivity (Figure 3C). Group C had a 3.387-fold [95% CI (1.159, 9.901), *p* = 0.026] increased risk for thromboembolic recurrence than group B, whose recurrence risk was 3.236 [95% CI (1.515, 6.914), *p* = 0.002] folds higher than that of group A.

**TABLE 3:** Univariable and multivariable Cox regression for a composite endpoint in patients with primary thrombotic antiphospholipid syndrome (ptAPS).

	HR	95% CI	*P* value
**Univariable Cox regression for the composite endpoint**			
Male gender	2.409	(1.271, 4.565)	0.007**
Age of onset	1.013	(0.995, 1.032)	0.162
Obesity	0.542	(0.131, 2.249)	0.399
Smoking	1.857	(1.017, 3.389)	0.044*
Hypertension	1.071	(0.529, 2.169)	0.849
Diabetes mellitus	1.795	(0.638, 5.052)	0.268
Dyslipidaemia	1.310	(0.552, 3.106)	0.540
Hyperhomocysteinemia	1.300	(0.668, 2.530)	0.440
Atherosclerosis	1.291	(0.619, 2.695)	0.496
Isolated anti-β2GP1	1.252	(0.446, 3.511)	0.670
Isolated LA	2.024	(0.988, 4.148)	0.054
aCL + anti-β2GP1	1.666	(0.771, 3.600)	0.194
anti-β2GP1 + LA	0.405	(0.098, 1.676)	0.212
aCL + LA	0.046	(0.000, 27.887)	0.346
Triple positive aPLs	0.647	(0.357, 1.170)	0.150
ANA positive	0.969	(0.530, 1.773)	0.918
ESR	0.997	(0.988, 1.006)	0.476
C-reactive protein	0.999	(0.994, 1.005)	0.853
IgG	0.990	(0.918, 1.067)	0.787
IgA	0.991	(0.938, 1.047)	0.746
IgM	0.846	(0.522, 1.374)	0.500
C3	1.145	(0.335, 3.915)	0.829
C4	0.223	(0.004, 12.581)	0.466
Aspirin	0.766	(0.392, 1.497)	0.436
Warfarin	0.509	(0.272, 0.952)	0.034*
Direct oral anticoagulants	1.191	(0.551, 2.576)	0.657
Corticosteroid	0.711	(0.329, 1.534)	0.384
Hydroxychloroquine	1.227	(0.669, 2.252)	0.509
Immunosuppressants	0.707	(0.276, 1.813)	0.470
**Multivariable Cox regression for composite endpoint**			
Male gender	2.254	(1.045, 4.858)	0.038*
Age of onset	1.005	(0.986, 1.025)	0.609
Smoking	1.155	(0.556, 2.396)	0.700
Diabetes mellitus	1.703	(0.560, 5.175)	0.348
Isolated LA	1.694	(0.819, 3.502)	0.155
Warfarin	0.513	(0.268, 0.985)	0.045*
**Final independent risk factors**			
Male gender	2.499	(1.316, 4.743)	0.005**
Warfarin	0.482	(0.257, 0.905)	0.023*

aPLs: antiphospholipid autoantibodies; aCL: anticardiolipin antibody; anti-β2GP1: anti-beta 2 glycoprotein I antibody; LA: lupus anticoagulant; ESR: erythrocyte sedimentation rate. *0.01 ≤ p < 0.05 and **0.001 ≤ p < 0.01.

**TABLE 4:** Univariable and multivariable Cox regression for thromboembolic recurrence in patients with primary thrombotic antiphospholipid syndrome (ptAPS).

	HR	95% CI	*P* value
**Univariable Cox regression for thromboembolic recurrence**			
Male gender	3.869	(1.747, 8.568)	0.001***
Age of onset	1.000	(0.978, 1.022)	0.979
Obesity	0.695	(0.166, 2.908)	0.618
Smoking	2.419	(1.230, 4.757)	0.010**
Hypertension	1.076	(0.488, 2.369)	0.856
Diabetes mellitus	1.742	(0.528, 5.745)	0.362
Dyslipidaemia	1.737	(0.719, 4.200)	0.220
Hyperhomocysteinemia	1.179	(0.551, 2.524)	0.672
Atherosclerosis	1.047	(0.433, 2.533)	0.919
Isolated anti-β2GP1	0.759	(0.181, 3.174)	0.705
Isolated LA	2.549	(1.187, 5.477)	0.016*
aCL + anti-β2GP1	1.519	(0.627, 3.679)	0.355
anti-β2GP1 + LA	0.252	(0.034, 1.841)	0.174
aCL + LA	0.046	(0.000, 56.486)	0.396
Triple positive aPLs	0.850	(0.437, 1.652)	0.631
ANA positive	0.846	(0.420, 1.702)	0.639
ESR	0.993	(0.983, 1.004)	0.227
C-reactive protein	0.998	(0.990, 1.006)	0.607
IgG	0.980	(0.898, 1.069)	0.647
IgA	0.985	(0.881, 1.102)	0.790
IgM	0.896	(0.533, 1.505)	0.677
C3	1.377	(0.345, 5.493)	0.651
C4	0.140	(0.002, 12.692)	0.393
Aspirin	0.834	(0.397, 1.753)	0.633
Warfarin	0.922	(0.425, 2.002)	0.838
Direct oral anticoagulants	0.908	(0.350, 2.354)	0.842
Corticosteroid	0.702	(0.291, 1.695)	0.432
Hydroxychloroquine	1.132	(0.574, 2.232)	0.720
Immunosuppressants	0.788	(0.277, 2.239)	0.655
**Multivariable Cox regression for thromboembolic recurrence**			
Male gender	3.062	(1.235, 7.591)	0.016*
Age of onset	0.991	(0.968, 1.015)	0.453
Smoking	1.465	(0.671, 3.199)	0.337
Diabetes mellitus	2.044	(0.576, 7.249)	0.268
Isolated LA	2.355	(1.066, 5.205)	0.034*
Warfarin	0.916	(0.408, 2.055)	0.831
**Final independent risk factors**			
Male gender	3.699	(1.659, 8.246)	0.001***
Isolated LA	2.236	(1.039, 4.811)	0.040*

aPLs: antiphospholipid autoantibodies; aCL: anticardiolipin antibody; anti-β2GP1: anti-beta 2 glycoprotein I antibody; LA: lupus anticoagulant; ESR: erythrocyte sedimentation rate. *0.01 ≤ p < 0.05, **0.001 ≤ p < 0.01, and ***p < 0.001.

## Discussion

To our knowledge, this is the first cohort study to investigate the relationship between the gender and prognosis of ptAPS patients with consensus aPLs in East Asia, which supported the findings of a previous case-control study (16). Considering thromboembolic distribution, limb ischemia was featured in male patients with ptAPS. Male gender and warfarin treatment were identified as independent risk factors for the composite endpoint including thromboembolic recurrence or all-cause death. As for thromboembolic recurrence alone, male gender and isolated LA positivity served as independent risk factors. Three recurrence risk groups were identified with gender and aPLs panel accordingly, among which male patients with isolated LA positivity had the highest risk of thromboembolic recurrence.

The sex differences in autoimmune diseases have been discussed for decades (17). So far, the female predominance in most systemic rheumatic diseases was partly attributed to estrogen-like molecules and epigenetics (18, 19). Since APS can be complicated with a great number of systemic rheumatic diseases and quite a few female patients present merely as obstetric events, evaluating sex differences in the whole APS population could be biased by female predominance. Hence, we recruited these patients with ptAPS to find sex differences in thromboembolic events and all-cause mortality. Complications leading to thrombophilia were also involved in the exclusion criteria, which might prevent some elderly patients from entering our cohort. In addition, differences in the age distribution pattern of APS between ethnic groups were suspected, since previous studies reported a younger APS population from East Asia than that from Europe (20, 21). A larger study should be expected to investigate the role of age in the prognosis of ptAPS.

In previously published case reports and case series, limb ischemia was relatively rare and sporadically reported in female patients with secondary APS (22–25). Our study identified that limb ischemia mostly occurred in male patients with ptAPS, which might be explained by potential endothelial dysfunction secondary to smoking and other cardiovascular risk factors such as hyperhomocysteinemia (26). Such discrepancies between male and female patients with ptAPS could also be a systemic bias due to the sex differences in cardiovascular morbidities in the general population, and further analysis from a larger cohort is required to clarify the role of traditional cardiovascular risk factors – especially serum C-reactive protein level, which was higher in male patients as we presented – in ptAPS. APS-associated chronic thromboembolic pulmonary hypertension (CTEPH), on the other hand, was a well-characterized subgroup with more frequent PE episodes than aPL-negative CTEPH, and male patients were reported to have a worse prognosis than their female counterparts (27, 28). Similarly, our data revealed that male patients with ptAPS tended to have more PE recurrence than their female counterparts.

Some studies demonstrated differences in thromboembolic lesion distribution between male and female patients with primary APS previously, but few of them investigated prognoses regarding thromboembolic recurrence and survival (9, 29). Our study was the first cohort to reveal the predilection of male patients with ptAPS to develop thromboembolic recurrence compared to their female counterparts. Such disparity might partly be attributed to worse compliance in male patients than female counterparts, which was particularly pronounced in smoking cessation, hyperhomocysteinemia correction, and unplanned medication discontinuation (30). The difference in gonadal steroid hormones might play a part in the male predominance in ptAPS thromboembolic recurrence. It has been recognized that exogenous gonadal steroid supplements, including estrogen as contraceptives or androgen as a muscle builder, were associated with an increased risk of arterial and venous thrombosis (31, 32). A case of ptAPS during treatment with aromatase inhibitors further confirmed the potential role of sex hormone imbalance in ptAPS development and thrombosis (33). Further investigations should be performed to explore the potential causes of such sex differences.

Since anti-β2GP1 is highly correlated with aCL (*p* < 0.001) and LA (*p* = 0.016), it is more suitable to classify aPLs status into 7 mutually exclusive categories as isolated aCL, isolated anti-β2GP1, isolated LA, aCL + anti-β2GP1, anti-β2GP1 + LA, aCL + LA, and triple positive in order to perform Cox regression. LA, especially isolated LA positivity, remained one of the most critical risk factors for thromboembolic events and recurrence in aPLs carriers and primary APS despite the discovery of novel aPLs subtypes, and our study reiterated the prognostic role of isolated LA positivity in the ptAPS population (34–37). Double positivity, especially anti-β2GP1 + LA, was more commonly seen in female than male patients with ptAPS and could contribute to the prognostic effect of sex, and triple positivity was at one time suspected to add to the risk of thromboembolic recurrence in a clinical trial of anticoagulants in patients with aPLs (38). Further analysis should be conducted to investigate the prognostic role of combined positivity vs. isolated positivity in ptAPS. For long-term thromboembolic prevention, standard-dose warfarin remained the best anticoagulation strategy so far, and our results displayed its benefit in extending survival (39). Considering immunomodulators, hydroxychloroquine might be effective as an adjuvant therapy according to a recently published pilot study (40).

Our study had some limitations. Though our cohort was the first one to investigate the impact of gender on ptAPS manifestations and prognosis, the sample size and follow-up period were still unsatisfactory, which might lead to some statistical bias. Since this prospective study was initiated in 2013, aPLs beyond the Sydney criteria including anti-phosphatidylserine/prothrombin antibodies were not tested due to technical issues. The monitoring of anticoagulation treatment failed to reach the minimum frequency of one time every three months in some patients.

## Conclusion

Our study demonstrated gender differences in ptAPS regarding lesion distribution, laboratory findings, and prognosis. Limb ischemia was more common in male patients. Male gender and warfarin treatment were identified as independent risk factors for the composite of thromboembolic recurrence and all-cause death. Considering thromboembolic recurrence alone, male gender and isolated LA positivity served as independent risk factors. The male patients with isolated LA positivity were at the highest risk for thromboembolic recurrence during follow-up and required extra attention.

## Data Availability Statement

The original contributions presented in the study are included in the article/Supplementary Material, further inquiries can be directed to the corresponding authors.

## Ethics Statement

The studies involving human participants were reviewed and approved by Institutional Review Board of PUMCH. The patients/participants provided their written informed consent to participate in this study.

## Author Contributions

XG, JZ, and YH: concept and design. YH, HL, WQ, and LD: data analysis. YH and HL: critical writing of the intellectual content. XG, JZ, ML, XZ, and SZ: final approval of the version to be published. All authors contributed to the article and approved the submitted version.

## Conflict of Interest

The authors declare that the research was conducted in the absence of any commercial or financial relationships that could be construed as a potential conflict of interest.

## Publisher’s Note

All claims expressed in this article are solely those of the authors and do not necessarily represent those of their affiliated organizations, or those of the publisher, the editors and the reviewers. Any product that may be evaluated in this article, or claim that may be made by its manufacturer, is not guaranteed or endorsed by the publisher.
