# Supplementary material for: Sex Differences in Clinical Characteristics and Prognosis in Primary Thrombotic Antiphospholipid Syndrome
Source: Front Cardiovasc Med. 2022 Jul 4;9:895098. doi: 10.3389/fcvm.2022.895098 (PMC9289156; doi:10.3389/fcvm.2022.895098)
Supplement: Supplementary file 1 [file Table_1.DOCX]

**Supplemental Table 1.** Demographic characteristics, treatment, and aPLs of ptAPS patients, categorized by presence of composite endpoint.

|  | Total | Composite Endpoint Met | Composite Endpoint Unmet | *P* value |
| --- | --- | --- | --- | --- |
| N | 154 | 45 | 109 | - |
| Male, n (%) | 80 (52%) | 30 (67%) | 50 (46%) | 0.019* |
| Age of onset, years | 36 (27, 51) | 37 (27, 60) | 35 (27, 46) | 0.310 |
| Disease duration, months | 1 (1, 10) | 1 (0, 24) | 1 (0, 7) | 0.426 |
| Cardiovascular risk factors |  |  |  |  |
| Body mass index, kg/m^2^ | 23.70 (21.41, 26.80) | 23.47 (20.01, 26.63) | 23.78 (21.92, 27.22) | 0.439 |
| Overweight, n (%) | 43 (28%) | 15 (33%) | 28 (26%) | 0.336 |
| Obesity, n (%) | 15 (9.7%) | 2 (4%) | 13 (12%) | 0.233 |
| Smoking, n (%) | 56 (36%) | 20 (44%) | 36 (33%) | 0.180 |
| Hypertension, n (%) | 34 (22%) | 10 (22%) | 24 (22%) | 0.978 |
| Diabetes mellitus, n (%) | 10 (6%) | 4 (9%) | 6 (6%) | 0.479 |
| Dyslipidaemia, n (%) | 18 (12%) | 6 (13%) | 12 (11%) | 0.683 |
| Hyperhomocysteinemia, n (%) | 35 (23%) | 12 (27%) | 23 (21%) | 0.454 |
| Atherosclerosis, n (%) | 27 (18%) | 9 (20%) | 18 (17%) | 0.605 |
| Treatment strategies |  |  |  |  |
| Aspirin, n (%) | 51 (33%) | 12 (27%) | 39 (36%) | 0.274 |
| Warfarin, n (%) | 106 (69%) | 28 (62%) | 78 (72%) | 0.255 |
| Direct oral anticoagulants, n (%) | 29 (19%) | 8 (18%) | 21 (19%) | 0.830 |
| Antiplatelet plus anticoagulant, n (%) | 38 (25%) | 9 (20%) | 29 (27%) | 0.387 |
| Corticosteroid, n (%) | 31 (20%) | 8 (18%) | 23 (21%) | 0.640 |
| Hydroxychloroquine, n (%) | 84 (54%) | 25 (56%) | 59 (54%) | 0.872 |
| Immunosuppressants, n (%) | 20 (13%) | 5 (11%) | 15 (14%) | 0.795 |
| Median follow-up time, months | 42 (23, 67) | 33 (11, 64) | 44 (26, 69) | 0.189 |
| aPLs categories |  |  |  |  |
| aCL, n (%) | 103 (67%) | 29 (64%) | 74 (68%) | 0.679 |
| anti-β2GP1, n (%) | 122 (79%) | 33 (73%) | 89 (82%) | 0.247 |
| LA, n (%) | 119 (77%) | 31 (69%) | 88 (81%) | 0.111 |
| Single positive, n (%) | 39 (25%) | 16 (36%) | 23 (21%) | 0.061 |
| Isolated aCL, n (%) | 2 (1%) | 2 (4%) | 0 | 0.084 |
| Isolated anti-β2GP1, n (%) | 14 (9%) | 4 (9%) | 10 (9%) | 1.000 |
| Isolated LA, n (%) | 23 (15%) | 10 (22%) | 13 (12%) | 0.103 |
| Double positive, n (%) | 40 (26%) | 10 (22%) | 30 (28%) | 0.495 |
| aCL + anti-β2GP1, n (%) | 19 (12%) | 8 (18%) | 11 (10%) | 0.187 |
| anti-β2GP1 + LA, n (%) | 14 (9%) | 2 (4%) | 12 (11%) | 0.354 |
| aCL + LA, n (%) | 7 (5%) | 0 | 7 (6%) | 0.107 |
| Triple positive, n (%) | 75 (49%) | 19 (42%) | 56 (51%) | 0.301 |

aPLs: antiphospholipid autoantibodies; aCL: anticardiolipin antibody; anti-β2GP1: anti-beta 2 glycoprotein I antibody; LA: lupus anticoagulant.
